# Photochemically produced SO_2_ in the atmosphere of WASP-39b

**DOI:** 10.1038/s41586-023-05902-2

**Published:** 2023-04-26

**Authors:** Shang-Min Tsai, Elspeth K. H. Lee, Diana Powell, Peter Gao, Xi Zhang, Julianne Moses, Eric Hébrard, Olivia Venot, Vivien Parmentier, Sean Jordan, Renyu Hu, Munazza K. Alam, Lili Alderson, Natalie M. Batalha, Jacob L. Bean, Björn Benneke, Carver J. Bierson, Ryan P. Brady, Ludmila Carone, Aarynn L. Carter, Katy L. Chubb, Julie Inglis, Jérémy Leconte, Michael Line, Mercedes López-Morales, Yamila Miguel, Karan Molaverdikhani, Zafar Rustamkulov, David K. Sing, Kevin B. Stevenson, Hannah R. Wakeford, Jeehyun Yang, Keshav Aggarwal, Robin Baeyens, Saugata Barat, Miguel de Val-Borro, Tansu Daylan, Jonathan J. Fortney, Kevin France, Jayesh M. Goyal, David Grant, James Kirk, Laura Kreidberg, Amy Louca, Sarah E. Moran, Sagnick Mukherjee, Evert Nasedkin, Kazumasa Ohno, Benjamin V. Rackham, Seth Redfield, Jake Taylor, Pascal Tremblin, Channon Visscher, Nicole L. Wallack, Luis Welbanks, Allison Youngblood, Eva-Maria Ahrer, Natasha E. Batalha, Patrick Behr, Zachory K. Berta-Thompson, Jasmina Blecic, S. L. Casewell, Ian J. M. Crossfield, Nicolas Crouzet, Patricio E. Cubillos, Leen Decin, Jean-Michel Désert, Adina D. Feinstein, Neale P. Gibson, Joseph Harrington, Kevin Heng, Thomas Henning, Eliza M.-R. Kempton, Jessica Krick, Pierre-Olivier Lagage, Monika Lendl, Joshua D. Lothringer, Megan Mansfield, N. J. Mayne, Thomas Mikal-Evans, Enric Palle, Everett Schlawin, Oliver Shorttle, Peter J. Wheatley, Sergei N. Yurchenko

**Affiliations:** 1 Atmospheric, Oceanic and Planetary Physics, Department of Physics, University of Oxford, Oxford, UK; 2 Department of Earth Sciences, University of California, Riverside, Riverside, CA USA; 3 Center for Space and Habitability, University of Bern, Bern, Switzerland; 4 Center for Astrophysics | Harvard & Smithsonian, Cambridge, MA USA; 5 Earth and Planets Laboratory, Carnegie Institution for Science, Washington, DC USA; 6 Department of Earth and Planetary Sciences, University of California, Santa Cruz, Santa Cruz, CA USA; 7 Space Science Institute, Boulder, CO USA; 8 University of Exeter, Exeter, UK; 9 Université de Paris Cité and Univ. Paris Est Creteil, CNRS, LISA, Paris, France; 10 Université Côte d’Azur, Observatoire de la Côte d’Azur, CNRS, Laboratoire Lagrange, Nice, France; 11 Institute of Astronomy, University of Cambridge, Cambridge, UK; 12 Jet Propulsion Laboratory, California Institute of Technology, Pasadena, CA USA; 13 Division of Geological and Planetary Sciences, California Institute of Technology, Pasadena, CA USA; 14 School of Physics, University of Bristol, Bristol, UK; 15 Department of Astronomy and Astrophysics, University of California, Santa Cruz, Santa Cruz, CA USA; 16 Department of Astronomy and Astrophysics, University of Chicago, Chicago, IL USA; 17 Department of Physics and Institute for Research on Exoplanets, Université de Montréal, Montreal, Quebec Canada; 18 School of Earth and Space Exploration, Arizona State University, Tempe, AZ USA; 19 Department of Physics and Astronomy, University College London, London, UK; 20 Space Research Institute, Austrian Academy of Sciences, Graz, Austria; 21 Centre for Exoplanet Science, University of St Andrews, St Andrews, UK; 22 Department of Physics & Astronomy, Johns Hopkins University, Baltimore, MD USA; 23 Laboratoire d’Astrophysique de Bordeaux, Université de Bordeaux, Pessac, France; 24 Leiden Observatory, University of Leiden, Leiden, the Netherlands; 25 SRON Netherlands Institute for Space Research, Leiden, the Netherlands; 26 Universitäts-Sternwarte München, Ludwig-Maximilians-Universität München, Munich, Germany; 27 Exzellenzcluster Origins, Munich, Germany; 28 Department of Earth & Planetary Sciences, Johns Hopkins University, Baltimore, MD USA; 29 Johns Hopkins Applied Physics Laboratory, Laurel, MD USA; 30 Indian Institute of Technology Indore, Indore, India; 31 Anton Pannekoek Institute for Astronomy, University of Amsterdam, Amsterdam, the Netherlands; 32 Planetary Science Institute, Tucson, AZ USA; 33 Department of Astrophysical Sciences, Princeton University, Princeton, NJ USA; 34 Laboratory for Atmospheric and Space Physics, University of Colorado Boulder, Boulder, CO USA; 35 School of Earth and Planetary Sciences (SEPS), National Institute of Science Education and Research (NISER), Homi Bhabha National Institute (HBNI), Odisha, India; 36 Department of Physics, Imperial College London, London, UK; 37 Max Planck Institute for Astronomy, Heidelberg, Germany; 38 Lunar and Planetary Laboratory, University of Arizona, Tucson, AZ USA; 39 Department of Earth, Atmospheric and Planetary Sciences, Massachusetts Institute of Technology, Cambridge, MA USA; 40 Kavli Institute for Astrophysics and Space Research, Massachusetts Institute of Technology, Cambridge, MA USA; 41 Astronomy Department and Van Vleck Observatory, Wesleyan University, Middletown, CT USA; 42 Maison de la Simulation, CEA, CNRS, Univ. Paris-Sud, UVSQ, Université Paris-Saclay, Gif-sur-Yvette, France; 43 Chemistry and Planetary Sciences, Dordt University, Sioux Center, IA USA; 44 NASA Goddard Space Flight Center, Greenbelt, MD USA; 45 Centre for Exoplanets and Habitability, University of Warwick, Coventry, UK; 46 Department of Physics, University of Warwick, Coventry, UK; 47 NASA Ames Research Center, Moffett Field, CA USA; 48 Department of Astrophysical and Planetary Sciences, University of Colorado Boulder, Boulder, CO USA; 49 Department of Physics, New York University Abu Dhabi, Abu Dhabi, United Arab Emirates; 50 Center for Astro, Particle, and Planetary Physics (CAP3), New York University Abu Dhabi, Abu Dhabi, United Arab Emirates; 51 School of Physics and Astronomy, University of Leicester, Leicester, UK; 52 Department of Physics & Astronomy, University of Kansas, Lawrence, KS USA; 53 INAF - Turin Astrophysical Observatory, Pino Torinese, Italy; 54 Institute of Astronomy, Department of Physics and Astronomy, KU Leuven, Leuven, Belgium; 55 School of Physics, Trinity College Dublin, Dublin, Ireland; 56 Planetary Sciences Group, Department of Physics and Florida Space Institute, University of Central Florida, Orlando, FL USA; 57 Department of Astronomy, University of Maryland, College Park, MD USA; 58 Infrared Processing and Analysis Center (IPAC), California Institute of Technology, Pasadena, CA USA; 59 Département d’Astronomie, Université de Genève, Sauverny, Switzerland; 60 Department of Physics, Utah Valley University, Orem, UT USA; 61 Steward Observatory, University of Arizona, Tucson, AZ USA; 62 Department of Physics and Astronomy, Faculty of Environment, Science and Economy, University of Exeter, Exeter, UK; 63 Instituto de Astrofísica de Canarias (IAC), Tenerife, Spain

**Keywords:** Exoplanets, Atmospheric chemistry, Giant planets

## Abstract

Photochemistry is a fundamental process of planetary atmospheres that regulates the atmospheric composition and stability^1^. However, no unambiguous photochemical products have been detected in exoplanet atmospheres so far. Recent observations from the JWST Transiting Exoplanet Community Early Release Science Program^2,3^ found a spectral absorption feature at 4.05 μm arising from sulfur dioxide (SO_2_) in the atmosphere of WASP-39b. WASP-39b is a 1.27-Jupiter-radii, Saturn-mass (0.28 *M*_J_) gas giant exoplanet orbiting a Sun-like star with an equilibrium temperature of around 1,100 K (ref. ^4^). The most plausible way of generating SO_2_ in such an atmosphere is through photochemical processes^5,6^. Here we show that the SO_2_ distribution computed by a suite of photochemical models robustly explains the 4.05-μm spectral feature identified by JWST transmission observations^7^ with NIRSpec PRISM (2.7*σ*)^8^ and G395H (4.5*σ*)^9^. SO_2_ is produced by successive oxidation of sulfur radicals freed when hydrogen sulfide (H_2_S) is destroyed. The sensitivity of the SO_2_ feature to the enrichment of the atmosphere by heavy elements (metallicity) suggests that it can be used as a tracer of atmospheric properties, with WASP-39b exhibiting an inferred metallicity of about 10× solar. We further point out that SO_2_ also shows observable features at ultraviolet and thermal infrared wavelengths not available from the existing observations.

## Abstract (web summary)

Observations from the JWST show the presence of a spectral absorption feature at 4.05 μm arising from SO_2_ in the atmosphere of the gas giant exoplanet WASP-39b, which is produced by photochemical processes and verified by numerical models.

## Main

JWST observed WASP-39b as part of its Transiting Exoplanet Community Early Release Science Program (ERS Program 1366), with the goal of explaining its atmospheric composition^2,3^. Data from the NIRSpec PRISM and G395H instrument modes showed a distinct absorption feature between 4.0 μm and 4.2 μm, peaking at around 4.05 μm, that atmospheric radiative–convective–thermochemical equilibrium models could not explain with metallicity and C/O values typically assumed of gas giant planets (1–100× solar and 0.3–0.9, respectively^8,9^). After excluding instrument systematics and stellar variability, a thorough search for gases has shown SO_2_ as a promising candidate with the best-fit absorption feature (see Methods), although ad hoc spectra with injected SO_2_ were used in the analysis.

**Extended Data Fig. 1:**
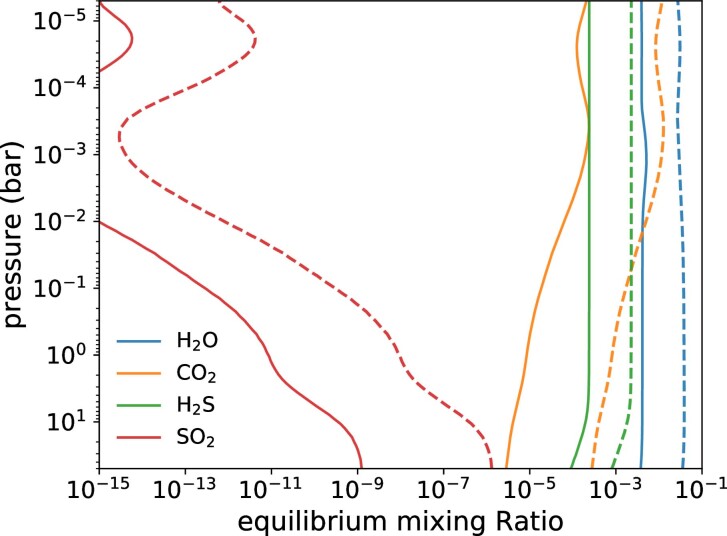
Chemical equilibrium abundances in the atmosphere of WASP-39b. VMR profiles of H_2_O (blue), CO_2_ (orange), H_2_S (green) and SO_2_ (red), as computed by FastChem (ref. ^38^) based on the morning terminator temperature profile, are given for 10× (solid lines) and 100× (dashed lines) solar metallicity.

Sulfur shares some chemical similarities with oxygen but uniquely forms various compounds with a wide range of oxidation states (−2 to +6 (ref. ^10^)). Although SO_2_ is ubiquitously outgassed and associated with volcanism on terrestrial worlds (for example, Earth, Venus and Jupiter’s satellite Io), the source of SO_2_ is fundamentally different on gas giants. Under thermochemical equilibrium, sulfur chiefly exists in the reduced form, such that H_2_S is the primary sulfur reservoir in a hydrogen/helium-dominated gas giant^11–14^. At the temperature of WASP-39b, the equilibrium mixing ratio of SO_2_ in the observable part of the atmosphere is less than about 10^−12^ for 10× solar metallicity and less than about 10^−9^ for even 100× solar metallicity (see Extended Data Fig. 1). This equilibrium abundance of SO_2_ is several orders of magnitude smaller than the values needed to produce the spectral feature observed by JWST (volume mixing ratios (VMRs) of 10^−6^–10^−5^)^8,9^. By contrast, under ultraviolet (UV) irradiation, SO_2_ can be oxidized from H_2_S as a photochemical product. H and OH radicals, generated by photolysis processes, are key to liberating SH radicals and atomic S from H_2_S and subsequently oxidizing them to SO and SO_2_. Although previous photochemical modelling studies have shown that substantial SO_2_ can be produced in hydrogen-rich exoplanet atmospheres in this way^5,6,13,15,16^, the extent to which such a model could reproduce the current WASP-39b observations remained unverified.

**Extended Data Fig. 2:**
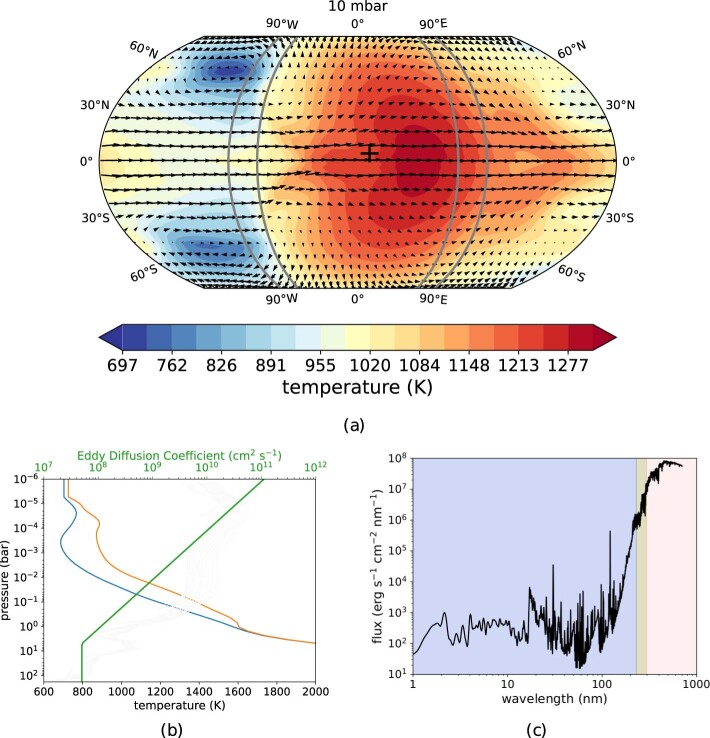
The temperature–wind map of the WASP-39b Exo-FMS GCM and input for 1D photochemical models. **a**, The colour scale represents temperature across the planet and arrows denote the wind direction and magnitude at 10 mbar. The ±10° longitudinal regions with respect to the morning and evening terminators are indicated with solid grey lines. The ‘+’ symbol denotes the sub-stellar point. **b**, 1D temperature–pressure profiles adopted from the morning and evening terminators averaging all latitudes and ±10° longitudes (regions enclosed by grey lines in **a**) and the *K*_zz_ profile (equation (2) and held constant below the 5-bar level) overlaying the root-mean-squared vertical wind multiplied by 0.1 scale height from the GCM (grey). The temperatures are kept isothermal from those at the top boundary of the GCM around 5 × 10^−5^ bar when extending to lower pressures (about 10^−8^ bar) for photochemical models. **c**, Input WASP-39 stellar flux at the surface of the star. The pink-shaded region indicates the optical wavelength range at which the stellar spectrum is directly measured, whereas the blue-shaded and green-shaded regions are those constructed from the Sun and HD 203244, respectively.

We have performed several independent, cloud-free 1D photochemical model calculations of WASP-39b using the ARGO, ATMO, KINETICS and VULCAN codes (see Methods for model details). All models included sulfur kinetic chemical networks and were run using the same vertical temperature–pressure profiles of the morning and evening terminators adopted from a 3D WASP-39b atmospheric simulation with the Exo-FMS general circulation model (GCM)^17^ (see Extended Data Fig. 2). The nominal models assumed a metallicity of 10× solar (ref. ^18^) with a solar C/O ratio of 0.55, whereas we explored the sensitivity to atmospheric properties.

**Fig. 1:**
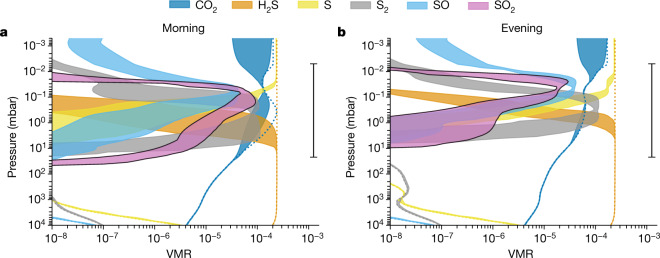
Simulated vertical distribution of sulfur species and CO_2_. **a**,**b**, The colour-shaded areas indicate the span (enclosed by the maximum and minimum values) of VMRs of CO_2_ (blue), SO_2_ (pink with black borders) and other key sulfur species (H_2_S, orange; S, yellow; S_2_, grey; SO, light blue) computed by an ensemble of photochemical models (ARGO, ATMO, KINETICS and VULCAN) for the morning (**a**) and evening (**b**) terminators. The thermochemical equilibrium VMRs are indicated by the dotted lines, with SO_2_ not within the *x*-axis range owing to its very low abundance in thermochemical equilibrium. The range bar on the right represents the main pressure ranges of the atmosphere investigated by JWST NIRSpec spectroscopy. Photochemistry produces SO_2_ and other sulfur species above the 1-mbar level with abundances several orders of magnitude greater than those predicted by thermochemical equilibrium.

The peak mixing ratios of the main sulfur species produced by the different photochemical models are largely consistent with each other to within an order of magnitude, as shown in Fig. 1. The SO_2_ mixing ratio profiles are highly variable with altitude and strongly peaked at 0.01–1 mbar with a value of 10–100 ppm. SO_2_ (along with CO_2_) is more favoured at the cooler morning terminator, at which H_2_S is less stable against reaction with atomic H at depth (with SO_2_ abundance peak of 50–90 ppm at the morning terminator and 15–30 ppm at the evening terminator). Although the peak SO_2_ abundance from the photochemical models is greater than that estimated from fitting to the PRISM and G395H data, which assumed vertically constant mixing ratios of about 1–10 ppm and about 2.5–4.6 ppm, respectively, the column-integrated number densities above 10 mbar are highly consistent (see Methods). Our models indicate that S, S_2_ and SO, which are precursors of SO_2_, also reach high abundances in the upper atmosphere above the pressure level at which H_2_S is destroyed. Nevertheless, they are not expected to manifest observable spectral features in the PRISM/G395H wavelength range.

**Fig. 2:**
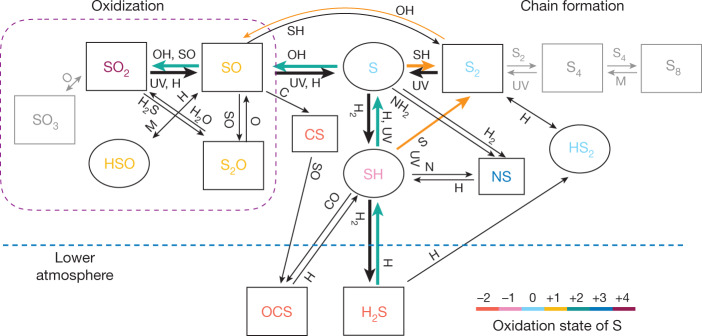
A simplified schematic of the chemical pathways of sulfur species. H_2_S, which is the stable sulfur-bearing molecule at thermochemical equilibrium in an H_2_ atmosphere, readily reacts with atomic H to form SH radicals and, subsequently, atomic S in the photochemical region (above about 0.1 mbar). Reaction of S with photochemically generated OH then produces SO, which is further oxidized to SO_2_. The thick arrows denote efficient reactions and M denotes any third body. Inefficient reactions and inactive paths in the temperature regime of WASP-39b are greyed out. The cyan arrows mark the main path from H_2_S to SO_2_, whereas the orange arrows mark the paths that are important at higher pressures. Sulfur species are colour-coded by the oxidation states of S. Rectangles indicate stable molecules, whereas ovals indicate free radicals.

The important pathways of sulfur kinetics in the atmosphere of WASP-39b from our models are summarized in Fig. 2. The photochemical production paths of SO_2_ from H_2_S around the SO_2_ peak are as follows:

1 $$\begin{array}{c}\,\,\,{{\rm{H}}}_{2}{\rm{O}}\,\mathop{\longrightarrow }\limits^{h\nu }\,{\rm{O}}{\rm{H}}+{\rm{H}}\\ \,\,\,{{\rm{H}}}_{2}{\rm{O}}+{\rm{H}}\,\longrightarrow \,{\rm{O}}{\rm{H}}+{{\rm{H}}}_{2}\\ \,\,\,{{\rm{H}}}_{2}{\rm{S}}+{\rm{H}}\,\longrightarrow \,{\rm{S}}{\rm{H}}+{{\rm{H}}}_{2}\\ \,\,\,{\rm{S}}{\rm{H}}+{\rm{H}}\,\longrightarrow \,{\rm{S}}+{{\rm{H}}}_{2}\\ \,\,\,{\rm{S}}+{\rm{O}}{\rm{H}}\,\longrightarrow \,{\rm{S}}{\rm{O}}+{\rm{H}}\\ \frac{\,\,\,{\rm{S}}{\rm{O}}+{\rm{O}}{\rm{H}}\,\longrightarrow \,{\rm{S}}{{\rm{O}}}_{2}+{\rm{H}}}{{\rm{net:}}\,{{\rm{H}}}_{2}{\rm{S}}+2{{\rm{H}}}_{2}{\rm{O}}\,\longrightarrow \,{\rm{S}}{{\rm{O}}}_{2}+3{{\rm{H}}}_{2}}\end{array}$$

Water photolysis in equation (1) is an important source of atomic H that initiates the pathway. The last step of oxidizing SO into SO_2_ is generally the rate-limiting step. The oxidization of SO and photolysis of SO_2_ account for the main sources and sinks of SO_2_, which lead to altitude-varying distribution that peaks around 0.1 mbar (see Extended Data Fig. 4). At high pressures with less available OH, reactions involving S_2_ become important in oxidizing S (see Methods). The growth of elemental sulfur allotropes beyond S_2_ effectively stops for temperatures higher than approximately 750 K (refs. ^5,6^).

**Fig. 3:**
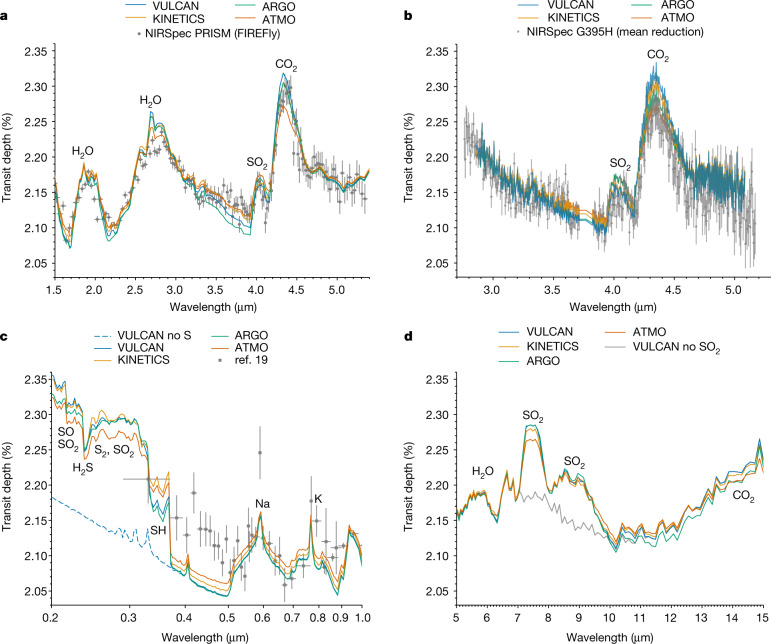
Terminator-averaged theoretical transmission spectra. We show the transmission spectra averaged over the morning and evening terminators generated from 1D photochemical model results. **a**, Comparison with the NIRSpec PRISM FIREFly reduction^8^. **b**, Comparison with the NIRSpec G395H weighted-mean reduction^9^. **c**, Comparison with the current HST and VLT/FORS2 optical wavelength data^19,37^. The models show pronounced features at UV wavelengths owing to sulfur species compared with the model without S-bearing species (dashed blue line). **d**, Predicted spectra across the MIRI LRS wavelength range, with SO_2_ removed from the VULCAN output shown in grey to indicate its contribution. All of the spectral data show 1*σ* error bars and the standard deviations averaged (unweighted) over all reductions are shown for the NIRSpec G395H data.

Figure 3 shows the morning/evening averaged transmission spectra resulting from the different photochemical models. All models are able to reproduce the strength and shape of the 4.05-μm SO_2_ feature seen in the NIRSpec PRISM and G395H modes. The scatter in the model spectra is on par with the uncertainties of the data and is attributed to the spread in the vertical VMR structure of SO_2_ and CO_2_ produced by each model (Fig. 1). Also shown in Fig. 3 are the predicted spectra in the MIRI LRS wavelength range (5–12 μm), which exhibit prominent SO_2_ features around 7.5 μm and 8.8 μm, as well as an upward slope redward of 12 μm owing to CO_2_. Furthermore, our models predict a strong UV (0.2–0.38 μm) transmission signal from the presence of S species: H_2_S, S_2_, SO_2_ and SH produce a sharp opacity gradient shortward of 0.38 μm (Extended Data Fig. 7), at which the room-temperature UV cross-sections are used except those at 800 K for SH. The discrepancy between the models and previous HST STIS and VLT/FORS2 observations^19^ (see Fig. 3) within 0.38–0.5 μm could potentially be because of enhanced UV opacities at high temperatures and/or aerosol particles. Further characterization of the sulfur species spectral features in the UV is promising with the scheduled HST/UVIS observation (Program 17162, principal investigators: Z. Rustamkulov and D. Sing).

**Fig. 4:**
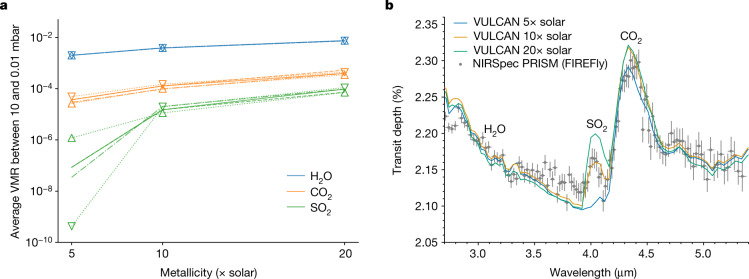
The metallicity trends and synthetic spectra with varying metallicity. **a**, The averaged VMR of H_2_O, CO_2_ and SO_2_ in the atmosphere between 10 and 0.01 mbar examined by transmission spectroscopy as a function of atmospheric metallicity. The nominal model is shown by solid lines, whereas the eddy diffusion coefficient (*K*_zz_) scaled by 0.1 and 10 are shown by dashed and dashed-dotted lines, respectively. The models with the whole temperature increased and decreased by 50 K are indicated by the upward-facing and downward-facing triangles connected by dotted lines, respectively. **b**, The morning and evening terminator-averaged theoretical transmission spectra with different metallicities (relative to solar value) compared with the NIRSpec observation. The error bars show 1*σ* standard deviations.

SO_2_ has recently been suggested as a promising tracer of metallicity in giant exoplanet atmospheres^16^. To test this and show trends in atmospheric properties, we have conducted sensitivity analysis on metallicity as well as temperature and vertical mixing using VULCAN (see Methods for details and further tests on C/O and stellar UV flux). Figure 4a summarizes these results for SO_2_, along with H_2_O and CO_2_, which are more commonly used as proxies for atmospheric metallicity^13,20–22^. Overall, the average abundance of SO_2_ in the pressure region relevant for such observation is not strongly sensitive to temperature or vertical mixing once SO_2_ has reached observable ppm levels and is mildly sensitive to C/O (see Extended Data Fig. 5). By contrast, SO_2_ shows either a similar or a stronger dependence on metallicity compared with H_2_O and CO_2_. This sensitivity to metallicity can be understood from the net reaction (equation (1)), in which it takes one molecule of H_2_S and two molecules of H_2_O to make one SO_2_. Although SO_2_ can be further oxidized into SO_3_, which requires extra oxygen, SO_3_ is rarely produced to an observable level in a H_2_-dominated atmosphere. Therefore, SO_2_ can be an ideal tracer of heavy-element enrichment for giant planets, with given constraints on the temperature and stellar far-ultraviolet (FUV) flux. The applicability of SO_2_ as a tracer of metallicity is further shown in Fig. 4b, in which the increase in the SO_2_ feature amplitude between 5× and 20× solar metallicity is much greater than that of CO_2_ and H_2_O. As such, retrieval analyses seeking to evaluate the atmospheric metallicity of warm giant exoplanets can substantially benefit from both CO_2_ and SO_2_ measurements.

Our results demonstrate the importance of considering photochemistry—and sulfur chemistry in particular—in warm exoplanet atmospheres when interpreting exoplanet atmospheric observations. Exoplanet photochemistry has been investigated using numerical models since the detection of an atmosphere on a transiting exoplanet^23,24^, followed by a diverse set of subsequent studies explaining the interplay of carbon, oxygen, nitrogen, hydrogen and sulfur (see, for example, ref. ^25^ for a review). It has been further pointed out that sulfur can affect other nonsulfur species, such as atomic H, CH_4_ and NH_3_ (refs. ^6,15^; also see Extended Data Fig. 6). Temperature trends in the photochemical production of sulfur species (Extended Data Fig. 10) in exoplanet atmospheres are potentially observable with features in the UV and infrared (Fig. 3 and Extended Data Fig. 7). At temperatures higher than that of WASP-39b, SH and SO may become relatively more abundant than SO_2_ (refs. ^6,13,15^). Observing these compositional variations with temperature in H_2_-dominated atmospheres, modulated by the atmospheric metallicity, could substantially improve our understanding of high-temperature chemical networks and atmospheric properties. The observational effort should also be complemented by a more accurate determination of key chemical reaction rate constants and UV cross-sections at the relevant temperatures (for example, refs. ^26,27^), as well as photochemical modelling development beyond 1D that includes horizontal transport (for example, refs. ^28–30^).

The accessibility of sulfur species in exoplanet atmospheres through the aid of photochemistry allows for a new window into planet-formation processes, whereas in the Solar System gas giants, the temperature is sufficiently low that sulfur is condensed out as either H_2_S clouds or together with NH_3_ as ammonium hydrosulfide (NH_4_SH) clouds^31^, making it more difficult to observe. Sulfur has been detected in protoplanetary disks^32^, in which it may be primarily in refractory form^33^, making it a reference element showing the metallicity contributions of accreted solid and gas^34–36^. Such efforts for warm giant exoplanets are now a possibility thanks to the observability of photochemically produced SO_2_. Thus, the detection of SO_2_ offers valuable insights into further atmospheric characterization and planet formation.

## Methods

### 4.05-μm feature

A list of gas species that have been compared with the 4.05-μm absorption feature in the transit observation of WASP-39b can be found in ref. ^8^. In particular, species with absorption features at similar wavelengths but are ruled out include H_2_S, HCN, HBr, PH_3_, SiO and SiO_2_. H_2_S and HCN absorb shortward of the feature at 4.05 μm, whereas SiO_2_ absorbs longward of that, and HBr, SiO and PH_3_ have wider absorption bands than the observed feature. Chemically, SiO and SiO_2_ are also expected to rain out at the temperature of WASP-39b and the solar elemental abundances have little bromine (Br/H ≈ 4 × 10^−10^). Ultimately, the injection tests of SO_2_ provide 2.7*σ* detection with NIRSpec PRISM (ref. ^8^) and 4.8*σ* with G395H (ref. ^9^).

### The temperature–pressure and eddy diffusion coefficient profiles derived from the Exo-FMS GCM

To provide inputs to the 1D photochemical models, a cloud-free WASP-39b GCM was run using the Exo-FMS GCM^17^. We computed the transmission spectra derived from our photochemical model results using gCMCRT (ref. ^40^) and the ExoAmes high-temperature SO_2_ line list^41^. System parameters were taken from ref. ^7^. We assume a 10× solar metallicity atmosphere in thermochemical equilibrium and use two-stream, correlated-*k* radiative transfer without optical and UV wavelength absorbers such as TiO, VO and Fe, which are assumed to have rained out from the atmosphere given the atmospheric temperatures of WASP-39b. The assumption about thermochemical equilibrium in radiative-transfer calculations will be discussed in the next section.

Although the temperatures of WASP-39b cross several condensation curves of sulfide clouds, such as Na_2_S and ZnS, the gas composition is not expected to be markedly affected. The elemental abundances of Na and Zn are less abundant than S (Na/S ≈ 0.13, Zn/S ≈ 0.0029), which would at most reduce approximately 20% of the total sulfur, similar to how oxygen is being sequestered in silicates and metals^42^. Furthermore, this full condensation is unlikely because sulfide condensates generally have high surface energies^43,44^ that inhibit efficient nucleation, consistent with the detection of gaseous sodium on WASP-39b (ref. ^8^).

The radius of WASP-39b is inflated notably and we assume an internal temperature of 358 K, taken from the relationship between irradiated flux and internal temperature found in ref. ^45^. Extended Data Fig. 2a shows the latitude–longitude map of the temperature at a pressure level of 10 mbar. The input to the photochemical models are the temperature–pressure profiles at the morning and evening limbs (Extended Data Fig. 2), which we compute by taking the average of the profiles over all latitudes and ±10° (as estimated from the opening-angle calculations from ref. ^46^) of the morning (western) and evening (eastern) terminators (that is, the region between the grey curves in Extended Data Fig. 2a. The cooler morning terminator as a result of the horizontal heat transport facilitated by the global circulation can be seen in the figure.

Vertical mixing in 1D chemical models is commonly parameterized by eddy diffusion. For exoplanets, the eddy diffusion coefficient (*K*_zz_) is in general a useful but loosely constrained parameter. For the 1D photochemical models used in this work, we assume that *K*_zz_ follows an inverse square-root dependence with pressure in the stratosphere (for example, ref. ^29^) as

2 $${K}_{{\rm{zz}}}({{\rm{cm}}}^{2}\,{{\rm{s}}}^{-1})=5\times 1{0}^{7}{\left(\frac{5{\rm{bar}}}{P}\right)}^{0.5}$$

and held constant below the 5-bar level in the convective zone. The eddy diffusion profile generally fits the root-mean-squared vertical wind multiplied by 0.1 scale height as the characteristic length scale from the GCM. The resulting *K*_zz_ profile is presented in Extended Data Fig. 2.

### Radiative feedback of disequilibrium composition

**Extended Data Fig. 3:**
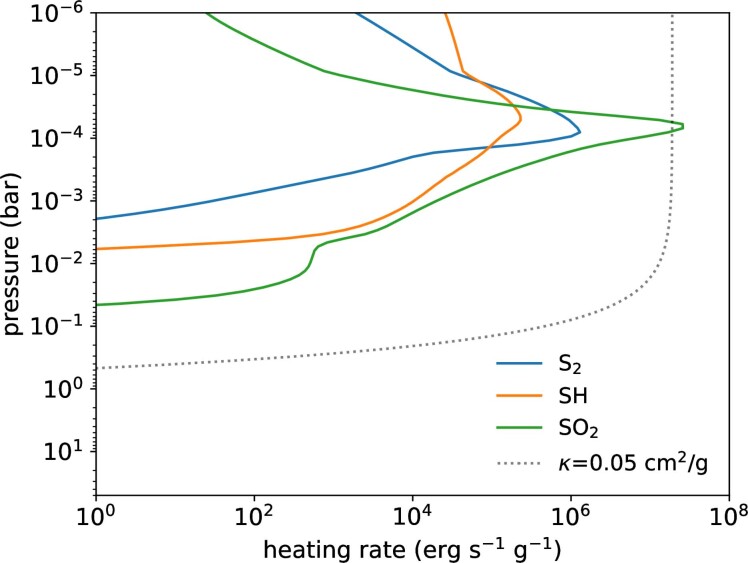
Shortwave radiative heating of sulfur species. Radiative heating rates (erg s^−1^ g^−1^) of SO_2_, SH and H_2_S to demonstrate their potential impact on the temperature structure. Heating owing to a vertically constant grey opacity of 0.05 cm^2^ g^−1^ is shown for comparison. All heating rates are integrated over 220–800 nm.

**Extended Data Fig. 4:**
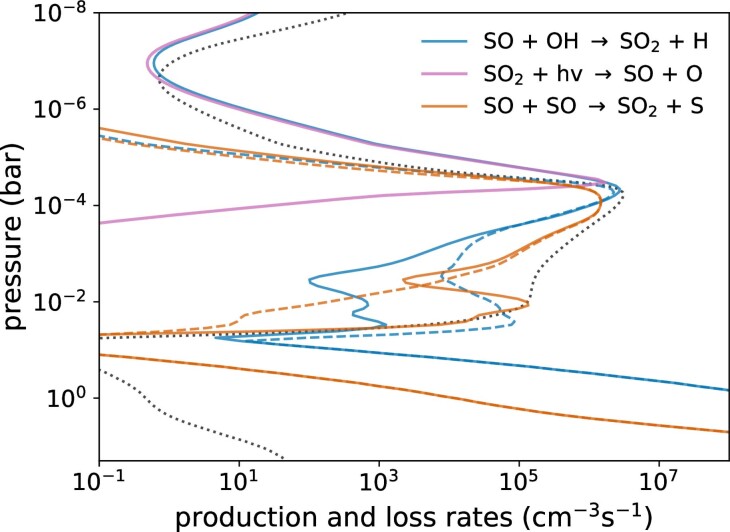
The main source and sink profiles of SO_2_ in our WASP-39b model. The reaction rates of the main sources and sinks of SO_2_ in the VULCAN morning-terminator model for WASP-39b. The dashed lines of the same colour are the corresponding reverse reactions and the dotted black line indicates the distribution profile (arbitrarily scaled) of SO_2_.

The temperature profiles adopted from the GCM assume chemical equilibrium abundances. To evaluate the radiative feedback from disequilibrium chemical abundances, we first performed self-consistent 1D calculations, coupling the radiative-transfer and photochemical-kinetics models using HELIOS (ref. ^47^) and VULCAN (ref. ^6^), for which the opacity sources in HELIOS include H_2_O, CH_4_, CO, CO_2_, NH_3_, HCN, C_2_H_2_, SH, H_2_S, SO_2_, Na, K, H^−^, CIA H_2_–H2 and H_2_–He (see references in ref. ^47^). Yet we found negligible differences between the temperature profile computed from equilibrium abundances and that from disequilibrium abundances. This is probably because water, as the predominant infrared opacity source, remains unaffected by disequilibrium processes. Meanwhile, a few opacities are missing in our radiative-transfer calculation. In particular, the opacity of SO_2_ (ref. ^48^) does not extend into the visible and UV wavelength range. Previous works^13,49^ indicated that SH and S_2_ have strong absorption in the UV–visible and can potentially affect the thermal structure. To quantify the radiative effect of these sulfur species, we calculated the shortwave heating rate with

3 $${c}_{{\rm{P}}}\frac{{\rm{d}}T}{{\rm{d}}t}=\frac{F{\kappa }_{i}\Delta {m}_{i}}{\Delta {m}_{{\rm{air}}}}$$

in which *c*_P_ is the specific heat capacity of the air, *F* is the stellar flux associated with the direct beam and Δ*m*_*i*_ and Δ*m*_air_ are the column mass of species *i* and air of an atmospheric layer, respectively. Extended Data Fig. 3 illustrates the shortwave heating owing to SH, S_2_ and SO_2_. Our estimate shows that SO_2_ contributed the most in our WASP-39b model, rather than SH and S_2_ being the main shortwave absorbers for atmospheres with solar-like metallicity^13,49^. The peak of heating owing to SO_2_ is comparable with a grey opacity of 0.05 cm^2^ g^−1^ over 220–800 nm and could potentially raise the temperatures around 0.1 mbar (the visible grey opacity for the irradiation of WASP-39b irradiation is about 0.005 cm^2^ g^−1^ (ref. ^50^)). Nevertheless, this heating effect does not change our main conclusions about photochemically forming SO_2_ on WASP-39b. As long as temperatures do not fall below roughly 750 K, at which sulfur allotrope formation starts to take over, SO_2_ is not too sensitive to temperature increases up to 100 K.

### The stellar spectrum of WASP-39

We require the high-energy spectral energy distribution (SED) of the WASP-39 host star as input to drive our set of photochemical models. However, as an inactive mid G-type star (*T*_eff_ = 5,485 ± 50 K; ref. ^51^) at a distance of 215 pc (Gaia DR3), WASP-39 is too faint for high-S/N UV spectroscopy with HST. To approximate the stellar radiation incident on WASP-39b, we created a custom stellar SED that combines direct spectroscopy of WASP-39 in the optical (with HST/STIS G430L and G750L modes; GO 12473, principal investigator: D. Sing) with representative spectra from analogous stars at shorter wavelengths.

Our approach to estimating the UV stellar SED was based on two factors: (1) in the near-ultraviolet (NUV; 2,300–2,950 Å), in which the flux is dominated by the photosphere, we chose a proxy with a similar spectral type to WASP-39 and (2) in the extreme ultraviolet (XUV) and FUV (1–2,300 Å), in which the stellar flux is dominated by chromospheric, transition region and coronal emission lines, we chose a proxy star with similar chromospheric activity indicators and used spectral type as a secondary consideration. In the NUV, we used HST/STIS E230M spectra of HD 203244, a relatively active (Ca II log(${R}_{{\rm{H}}{\rm{K}}}^{{\prime} }$) = −4.4 (ref. ^52^)), nearby (that is, unreddened, *d* = 20.8 pc; Gaia DR2), G5 V star (*T*_eff_ = 5,480 K (ref. ^53^)) from the STARCat archive^54^. Although HD 203244 is a suitable proxy at photospheric wavelengths, WASP-39 is a relatively old (about 7 Gyr) star with low chromospheric activity (log(${R}_{{\rm{H}}{\rm{K}}}^{{\prime} }$) = −4.97 ± 0.06) and a long rotation period (*P*_rot_ = 42.1 ± 2.6 days; ref. ^51^), suggesting substantially lower high-energy flux than HD 203244. Therefore, we elected to use a lower-activity G-type star, the Sun, at wavelengths shorter than 2,300 Å. The Sun has high-quality archival data available across the UV and X-rays and similar chromospheric activity to WASP-39 (the average solar Ca II log(${R}_{{\rm{H}}{\rm{K}}}^{{\prime} }$) value is −4.902 ± 0.063 and ranges from approximately −4.8 to −5.0 from solar maximum to solar minimum^55,56^). With the components in hand, we first corrected the observed STIS spectra of WASP-39 for interstellar dust extinction of *E*(*B* − *V*) = 0.079 (ref. ^57^) using a standard *R*_*V*_ = 3.1 interstellar reddening curve^58^ and then interpolated all spectra onto a 0.5-Å-pixel^−1^ grid. The NUV spectrum of HD 203244 was scaled to the reddening-corrected WASP-39 observations in the overlap region between 2,900 and 3,000 Å and the XUV + FUV spectrum of the quiet Sun^59^ was scaled to the blue end of the combined SED. The flux scaling between two spectral components is defined as ((*F*_ref_ − *α* × *F*_proxy_)/*σ*_ref_)^2^ in the overlap region, in which ‘proxy’ is the spectrum being scaled, ‘ref’ is the spectrum to which we are scaling and *α* is the scale factor applied to the proxy spectrum. *α* is varied until the above quantity is minimized (*α* = 2.04 × 10^−16^ and 7.58 × 10^−3^ for the FUV and NUV components, respectively). The final combined spectrum was convolved with a 2-Å full width at half maximum Gaussian kernel and wavelengths longer than 7,000 Å were removed to avoid the near-infrared fringing in the STIS G750L mode. We show the stellar spectrum at the surface of the star used for our photochemical models in Extended Data Fig. 2.

We compared our estimated SED for WASP-39 against archival GALEX observations from Shkolnik^60^, who found the NUV (1,771–2,831 Å) flux density to be 168.89 μJy, or an average NUV spectral flux of *F*_*λ*_ = 9.8 × 10^−16^ erg cm^−2^ s^−1^ Å^−1^ at 2,271 Å. Correcting this value by the average extinction correction in the GALEX NUV bandpass, a factor of 1.79, and comparing it with the average flux of our estimated SED over the same spectral range (1.66 × 10^−15^ erg cm^−2^ s^−1^ Å^−1^), we find the agreement between the GALEX measurement of WASP-39 and our stellar proxy to be better than 6%.

### Simulated transmission spectra from gCMCRT

To post-process the 1D photochemical model output and produce transmission spectra, we use the 3D Monte Carlo radiative-transfer code gCMCRT^40^.

For processing 1D columns, gCMCRT uses 3D spherical geometry but with a constant vertical profile across the globe in latitude and longitude. In this way, spectra from 1D outputs can be computed. We process the morning and evening terminator vertical 1D chemical profiles of each photochemical model separately, taking the average result of the two transmission spectra to produce the final spectra that are compared with the observational data.

In the transmission spectra model, we use opacities generated from the following line lists: H_2_O (ref. ^61^), OH (ref. ^62^), CO (ref. ^63^), CO_2_ (ref. ^64^), CH_4_ (ref. ^65^), CH_3_ (ref. ^66^), HCN (ref. ^67^), C_2_H_2_ (ref. ^68^), C_2_H_4_ (ref. ^69^), C_2_H_6_ (ref. ^70^), C_4_H_2_ (ref. ^70^), C_2_ (ref. ^71^), CN (ref. ^72^), CH (ref. ^73^), SO_2_ (ref. ^41^), SH (ref. ^48^), SO (ref. ^74^), H_2_S (ref. ^75^), NO (ref. ^76^), N_2_O (ref. ^76^), NO_2_ (ref. ^76^), HCl (ref. ^70^), Na (ref. ^77^), K (ref. ^77^).

### Description of photochemical models

We use the following 1D thermo-photochemical models to produce the steady-state chemical abundance profiles for the terminators of WASP-39b. All models assume cloud-free conditions and adopt the same temperature profiles, stellar UV flux, eddy diffusion coefficient profile (Extended Data Fig. 2) and zero-flux (closed) boundary conditions. A zenith angle of 83° (an effective zenith angle that matches the terminator-region-mean actinic flux for near-unity optical depth) is assumed for the terminator photochemical modelling.

#### VULCAN

The 1D kinetics model VULCAN treats thermochemical^78^ and photochemical^6^ reactions. VULCAN solves the Eulerian continuity equations, including chemical sources/sinks, diffusion and advection transport, and condensation. We applied the C-H-N-O-S network (https://github.com/exoclime/VULCAN/blob/master/thermo/SNCHO_photo_network.txt) for reduced atmospheres containing 89 neutral C-bearing, H-bearing, O-bearing, N-bearing and S-bearing species and a total of 1,028 thermochemical reactions (that is, 514 forward–backward pairs) and 60 photolysis reactions. The sulfur allotropes are simplified into a system of S, S_2_, S_3_, S_4_ and S_8_. The sulfur kinetics data are drawn from the NIST and KIDA databases, as well as modelling^5,79^ and ab initio calculations published in the literature (for example, ref. ^80^). For simplicity and cleaner model comparison, the temperature-dependent UV cross-sections^6^ are not used in this work. The pathfinding algorithm described in ref. ^81^ is used to identify the important chemical pathways. We note that the paths presented in this study are mainly based on VULCAN output (see Extended Data Table 1). Although detailed reactions might differ between different photochemical models, the main paths remain robust.

#### KINETICS

The KINETICS 1D thermo-photochemical transport model^42^ uses the Caltech/JPL KINETICS model^82,83^ to solve the coupled 1D continuity equations describing the chemical production, loss and vertical transport of atmospheric constituents of WASP-39 b. The model contains 150 neutral C-bearing, H-bearing, O-bearing, N-bearing, S-bearing and Cl-bearing species that interact with each other through a total of 2,350 reactions (that is, 1,175 forward–reverse reaction pairs). These reactions have all been fully reversed through the thermodynamic principle of microscopic reversibility^84^, such that the model would reproduce thermochemical equilibrium in the absence of transport and external energy sources, given sufficient integration time. The chemical reaction list involving C-bearing, H-bearing, O-bearing and N-bearing species is taken directly from ref. ^22^. Included for the first time here are 41 sulfur and chlorine species: S, S(1D), S_2_, S_3_, S_4_, S_8_, SH, H_2_S, HS_2_, H_2_S_2_, CS, CS_2_, HCS, H_2_CS, CH_3_S, CH_3_SH, SO, SO_2_, SO_3_, S_2_O, HOSO_2_, H_2_SO_4_ (gas and condensed), OCS, NS, NCS, HNCS, Cl, Cl_2_, HCl, ClO, HOCl, ClCO, ClCO_3_, ClS, ClS_2_, Cl_2_S, ClSH, OSCl, ClSO_2_ and SO_2_Cl_2_. The thermodynamic data of several chlorine-bearing and sulfur-bearing species are not available in the previous literature and we performed ab initio calculations for these species. We first carried out electronic-structure calculations at the CBS-QB3 level of theory using Gaussian 09 (ref. ^85^) to determine geometric conformations, energies and vibrational frequencies of the target molecules. Then the thermodynamic properties of these molecules were calculated by Arkane (ref. ^86^), a package included in the open-source software RMG v3.1.0 (refs. ^87,88^), with atomic-energy corrections, bond corrections and spin–orbit corrections, based on the CBS-QB3 level of theory as the model chemistry. The reaction rate coefficients and photolysis cross-sections for these S and Cl species are derived from Venus studies^89–94^, interstellar medium studies^95^, Io photochemical models^96,97^, Jupiter cometary-impact models^98,99^, the combustion-chemistry literature^100–103^, terrestrial stratospheric compilations^104,105^ and numerous individual laboratory or computational kinetics studies (such as refs. ^106–110^).

#### ARGO

The 1D thermochemical and photochemical kinetics code ARGO originally^111,112^ used the Stand2019 network for neutral hydrogen, carbon, nitrogen and oxygen chemistry. ARGO solves the coupled 1D continuity equation including thermochemical-photochemical reactions and vertical transport. The Stand2019 network was expanded by Rimmer et al. ^113^ by updating several reactions, incorporating the sulfur network developed by ref. ^15^, and supplementing it with reactions from refs. ^93,114^, to produce the Stand2020 network. The Stand2020 network includes 2,901 reversible reactions and 537 irreversible reactions, involving 480 species composed of H, C, N, O, S, Cl and other elements.

#### ATMO

The C-H-N-O chemical kinetics scheme from ref. ^115^ is implemented by ref. ^116^ in the standard 1D atmosphere model ATMO, which solves for the chemical disequilibrium steady state. As of the time of writing of this article, the sulfur kinetic scheme of ATMO, derived from applied combustion models, is still at the development and validation stage. Hence, for WASP-39b, we performed ATMO with the C-H-N-O-S thermochemical network from VULCAN (ref. ^6^) along with the photochemical scheme from ref. ^117^ (an update of the native photochemical scheme from ref. ^115^), with another 71 photolysis reactions of H_2_S, S_2_, S_2_O, SO, SO_2_, CH_3_SH, SH, H_2_SO and COS.

### Sensitivity tests

We examine the sensitivity of our chemical outcomes to essential atmospheric properties using VULCAN. For models with various metallicity and C/O ratios, we explore the sensitivity to temperature and vertical mixing by systematically varying the temperature–pressure and eddy diffusion coefficient profiles. Specifically, the temperature throughout the atmosphere is shifted by 50 K and the eddy diffusion coefficients are multiplied/divided by 10. These variations span a range comparable with the temperature differences among radiative transfer models^47^ and the uncertainties in parameterizing vertical mixing with eddy diffusion coefficients^118,119^. On our choice of internal heat, we have further conducted tests with different internal temperatures and found that the compositions above 1 bar are not sensitive to internal temperature, because the quench levels of the main species are at higher levels given the adopted eddy diffusion coefficient. We have also verified that the temperature above the top boundary of the GCM (about 5 × 10^−5^ bar; Extended Data Fig. 2) does not affect the composition below.

**Extended Data Fig. 5:**
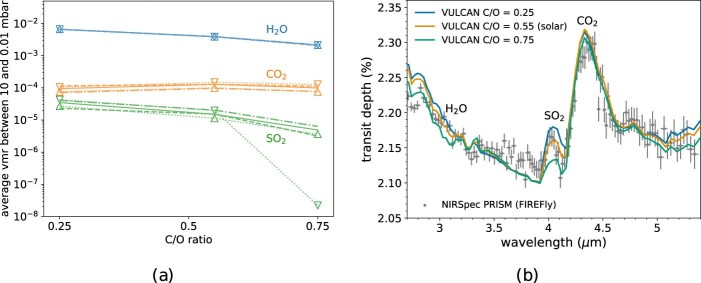
The C/O trends and synthetic spectra. Same as Fig. 4 but as a function of C/O ratio at 10× solar metallicity. **a**, The averaged VMR of H_2_O, CO_2_ and SO_2_ between 10 and 0.01 mbar as a function of C/O ratio, in which the solar C/O is 0.55. The nominal model is shown by solid lines, whereas the eddy diffusion coefficient (*K*_zz_) scaled by 0.1 and 10 are shown by dashed and dashed-dotted lines, respectively. The models for which the whole temperature increased and decreased by 50 K are indicated by the upward-facing and downward-facing triangles connected by dotted lines, respectively. **b**, The morning and evening terminator-averaged theoretical transmission spectra with different C/O ratios compared with the NIRSpec PRISM observation. The error bars show 1*σ* standard deviations.

**Extended Data Fig. 6:**
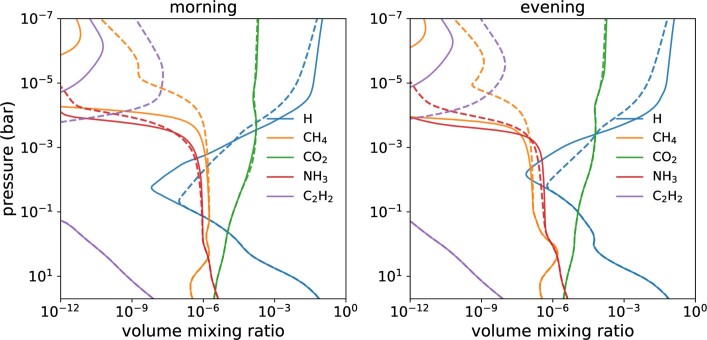
The impact of sulfur on other nonsulfur species. VMR profiles of some species in our WASP-39b model that exhibit differences from VULCAN including sulfur kinetics (solid lines) and without sulfur kinetics (dashed lines).

Sensitivity to C/O is summarized in Extended Data Fig. 5, in which the nominal model has a C/O ratio of 0.55, as in the main text. The averaged abundance of both SO_2_ and H_2_O in the pressure region relevant for transmission spectrum observations show similar dependencies on C/O, decreasing by a few factors as the C/O increased from sub-solar (0.25) to super-solar (0.75) values. The averaged abundance of SO_2_ is not very sensitive to temperature and vertical mixing either, except for C/O = 0.75, for which the SO_2_ concentration is at roughly the ppm level, similar to what is found in Fig. 4.

Finally, we performed sensitivity tests to the UV irradiation—the ultimate energy source of photochemistry. We first tested the sensitivity to the assumed stellar spectra by performing the same models with the solar spectrum (close to WASP-39) and found negligible differences in the photochemical results. Because the UV spectrum shortward of 295 nm is constructed from stellar proxies rather than directly measured, we then focused on varying the stellar flux in the FUV (1–230 nm) and NUV (230–295 nm) separately. Extended Data Fig. 8 shows that the resulting sulfur species abundances are almost identical when the UV flux is reduced by a factor of 10, broadly consistent with what Zahnle et al.^5^ suggested that the photochemical destruction of H_2_S only becomes photon-limited when the stellar UV flux is reduced by about two orders of magnitude (for a directly imaged gas giant). On the other hand, although SO and SO_2_ are not sensitive to increased NUV, they are substantially depleted with increased FUV. This is because the photodissociation of SO and SO_2_ mainly operates in the FUV and the enhanced FUV can destroy SO and SO_2_, even with the same amount of available OH radicals.

### Spectral effects of assuming a vertically uniform SO_2_ distribution

Minor species commonly have VMR varying with altitude in the observable region of the atmosphere, especially those produced or destroyed by photochemistry. Extended Data Fig. 9 demonstrates that assuming a vertically constant VMR of SO_2_ can lead to underestimating its abundances by about an order of magnitude. This is verified by comparing the column-integrated number density from the pressure level relevant for transmission spectroscopy. For example, the terminator-averaged column-integrated number density of SO_2_ above 10 mbar by VULCAN is about 1.4 × 10^19^ molecules cm^−2^, which is equal to a vertically uniform SO_2_ with a concentration around 4 ppm. Hence modelling frameworks that assume vertically uniform composition should be treated with caution and would benefit from comparisons with photochemical models, especially for photochemically active species that can exhibit large vertical gradients.

### Opacities of sulfur species

**Extended Data Fig. 7:**
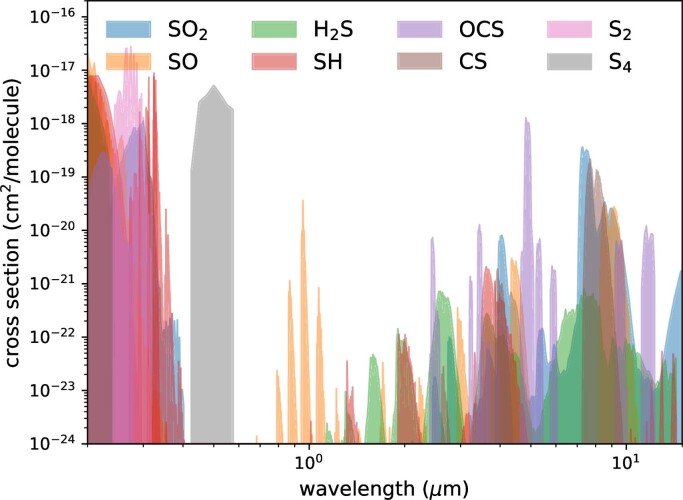
The opacities of several sulfur species. Opacities of several sulfur species at 1,000 K and 1 mbar, except that those in the UV and of OCS are at room temperature. The opacities in the infrared are binned down to *R* ≈ 1,000 for clarity.

**Extended Data Fig. 8:**
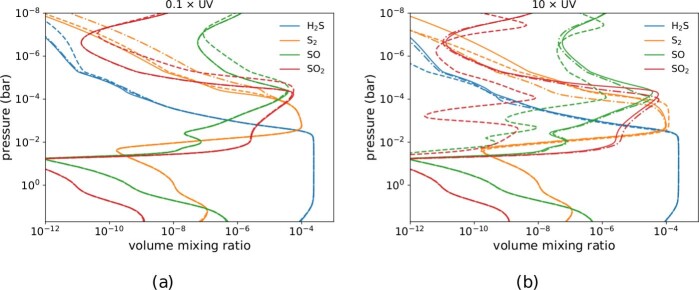
The main sulfur species abundances with reduced and enhanced UV irradiation. VMR profiles of the main sulfur species in the VULCAN morning-terminator model with 0.1× (**a**) and 10× (**b**) UV. Our nominal model is shown by solid lines for comparison, whereas the model with varying FUV (1–230 nm) is shown by the dashed lines and that with varying NUV (230–295 nm) is shown by dashed-dotted lines.

**Extended Data Fig. 9:**
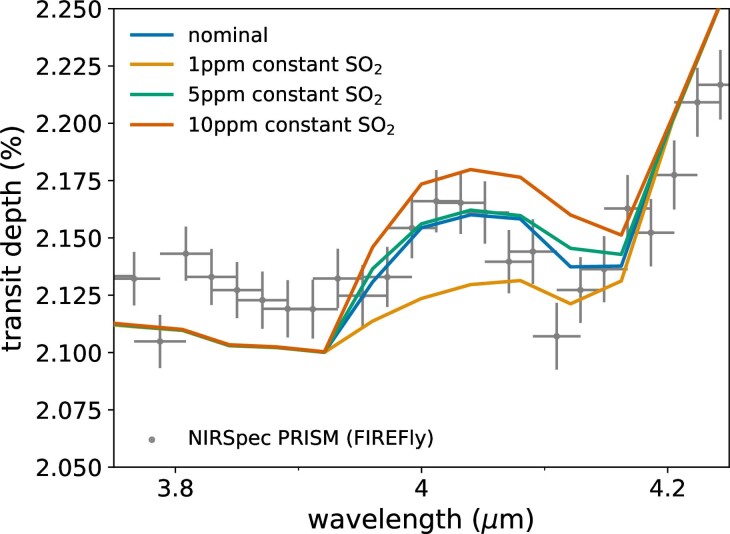
The effects of assuming a vertically uniform distribution of SO_2_. Terminator-averaged theoretical transmission spectra generated from abundance distribution computed by the photochemical model VULCAN compared with assuming constant 1, 5 and 10 ppm of SO_2_. As before, the NIRSpec PRISM observation is shown with 1*σ* error bars.

The opacities of sulfur species illustrated in Extended Data Fig. 7 are compiled from UV cross-sections and infrared line lists. The room-temperature UV cross-sections are taken from the Leiden Observatory database^120^ (http://home.strw.leidenuniv.nl/~ewine/photo). The infrared opacities include SO_2_ (ref. ^121^), H_2_S (refs. ^122,48^), CS (ref. ^123^) and a newly computed high-temperature line list for SO (ref. ^74^). The opacity from OCS (ref. ^124^) is only available up to room temperature at present, hence its coverage is probably incomplete in our region of interest.

### Alternative SO_2_ production pathways

S_2_ formation can compete with SO_2_ production, as we will explore in detail in the next section. On WASP-39b, reactions involving S_2_ are found to be important in oxidizing S at high pressures at which less OH is available. S and SH would first react to form S_2_ by SH + S → H + S_2_ before getting oxidized through S_2_ + OH → SO + SH. The scheme is similar to that in equation (1) except SH and S_2_ play the role of the catalyst to oxidize S into SO, whereas SO can also self-react to form SO_2_ in this regime (references of important reactions are listed in Extended Data Table 1).

### Implications of observing sulfur photochemistry

The temperature of WASP-39b resides within the sweet spot of producing SO_2_ (ref. ^16^). Previous photochemical modelling works suggested that, at lower temperatures, sulfur allotropes would be favoured over SO_2_, whereas SH can prevail at higher temperatures^5,6^. Here we briefly explain the general temperature trends of sulfur photochemical products.

**Extended Data Fig. 10:**
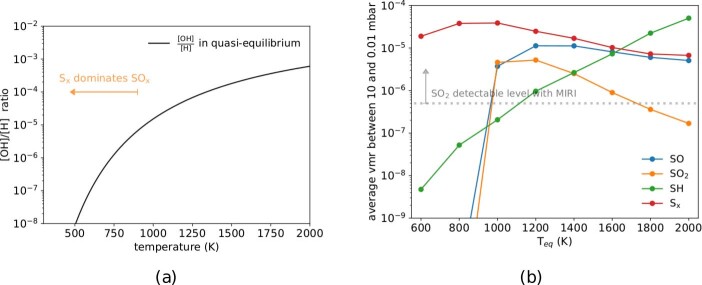
The OH to H ratio and the temperature trends for sulfur molecules produced by photochemistry. **a**, ${k}_{{{\rm{H}}}_{2}{\rm{O}}}/{k}_{{{\rm{H}}}_{2}{\rm{O}}}^{{\prime} }\times {\rm{O/H}}$ as a proxy of OH to H ratio at 10× solar metallicity, in which ${k}_{{{\rm{H}}}_{2}{\rm{O}}}$ and ${k}_{{{\rm{H}}}_{2}{\rm{O}}}^{{\prime} }$ are the forward and backward rate constants of H_2_O + H → OH + H_2_, respectively. When OH becomes scarce relative to H as temperature decreases, the chain-forming path (does not require OH) is favoured over the oxidization path (requires OH). **b**, The average VMR between 10 and 0.01 mbar as a function of planetary equilibrium temperature with temperature profiles adopted from ref. ^39^ (see text for the setup). The dotted grey line marks approximately the required SO_2_ concentration to be detectable with WASP-39b parameters. S_*x*_ denotes the allotropes S_2_ and S_8_ and SO_*x*_ denotes the oxidized species SO and SO_2_.

After S is liberated from H_2_S, sulfur can follow either the oxidization or the chain polymerization paths, as illustrated in Fig. 2. The competing of the two paths is essentially controlled by the abundance of the oxidizing radical OH relative to atomic H. We can estimate the OH to H ratio by assuming that OH is in quasi-equilibrium with H_2_O, that is, ${k}_{{{\rm{H}}}_{2}{\rm{O}}}[{{\rm{H}}}_{2}{\rm{O}}][{\rm{H}}]={k}_{{{\rm{H}}}_{2}{\rm{O}}}^{{\prime} }[{\rm{OH}}][{{\rm{H}}}_{2}]$, in which ${k}_{{{\rm{H}}}_{2}{\rm{O}}}$ and ${k}_{{{\rm{H}}}_{2}{\rm{O}}}^{{\prime} }$ are the forward and backward rate constants of H_2_O + H → OH + H_2_, respectively. Then, ${\rm{[OH]/[H]}}\approx 2\frac{{k}_{{{\rm{H}}}_{2}{\rm{O}}}}{{k}_{{{\rm{H}}}_{2}{\rm{O}}}^{{\prime} }}\times {\rm{O}}/{\rm{H}}$, because most of the O is in H_2_O. Extended Data Fig. 10a shows that the [OH]/[H] ratio strongly depends on temperature. When the temperature drops below about 750 K, the scarcity of OH makes S preferably react with SH to form S_2_. SO and SO_2_ could only be produced at higher altitudes, at which more OH is available from water photolysis (for example, refs. ^5,6^).

We further perform photochemical calculations using VULCAN with a grid of temperature profiles across planetary equilibrium temperatures 600–2,000 K, adopted from the 1D radiative–convective equilibrium models applied in ref. ^39^, in which an internal temperature of 100 K with perfect heat redistribution and gravity *g* = 1,000 cm s^−2^ are assumed. Apart from the thermal profiles, we keep the rest of the planetary parameters the same as the WASP-39b model in this work, including stellar UV irradiation. Extended Data Fig. 10b reveals the observation of sulfur photochemistry on other irradiated exoplanets, summarizing the averaged abundances of the key sulfur molecules produced by photochemistry as a function of equilibrium temperature. For 10× solar metallicity, the sweet-spot temperature for producing observable SO_2_ is 1,000 K ≲ *T*_eq_ ≲ 1,600 K. For *T*_eq_ ≲ 1,000 K, SO_2_ production below the 0.01-mbar level ceased and S_*x*_ (sulfur allotropes; mainly S_2_ and S_8_ here) is more favoured. For *T*_eq_ ≳ 1,600 K, SH becomes the predominant sulfur-bearing molecular (apart from atomic S) around mbar levels. Although observing SH is challenging in the infrared, it can potentially be identified in the near-UV (300–400 nm)^125^.

## Online content

Any methods, additional references, Nature Portfolio reporting summaries, source data, extended data, supplementary information, acknowledgements, peer review information; details of author contributions and competing interests; and statements of data and code availability are available at 10.1038/s41586-023-05902-2.

### Supplementary information

Peer Review File

## Figures and Tables

**Extended Data Table 1:**
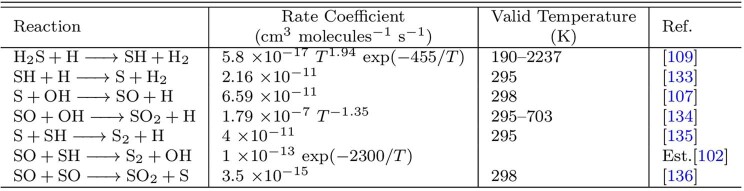
Important reactions for SO_2_ production

List of selected reactions relevant for SO_2_ production in the VULCAN model of WASP-39b.

## Data Availability

The data used in this paper are associated with JWST ERS Program 1366 and are available from the Mikulski Archive for Space Telescopes (https://mast.stsci.edu), which is operated by the Association of Universities for Research in Astronomy, Inc., under NASA contract NAS 5-03127 for JWST. The chemical networks and abundance output of the photochemical models (ARGO, ATMO, KINETICS and VULCAN) presented in this study are available at 10.5281/zenodo.7542781.

## Code availability

The codes VULCAN and gCMCRT used in this work to simulate composition and produce synthetic spectra are publicly available: VULCAN^6,78^ (https://github.com/exoclime/VULCAN); gCMCRT^40^ (https://github.com/ELeeAstro/gCMCRT).
